# ZikaVR: An Integrated Zika Virus Resource for Genomics, Proteomics, Phylogenetic and Therapeutic Analysis

**DOI:** 10.1038/srep32713

**Published:** 2016-09-16

**Authors:** Amit Kumar Gupta, Karambir Kaur, Akanksha Rajput, Sandeep Kumar Dhanda, Manika Sehgal, Md. Shoaib Khan, Isha Monga, Showkat Ahmad Dar, Sandeep Singh, Gandharva Nagpal, Salman Sadullah Usmani, Anamika Thakur, Gazaldeep Kaur, Shivangi Sharma, Aman Bhardwaj, Abid Qureshi, Gajendra Pal Singh Raghava, Manoj Kumar

**Affiliations:** 1 Bioinformatics Centre, Institute of Microbial Technology, Council of Scientific and Industrial Research (CSIR), Sector 39A, Chandigarh, 160036, India

## Abstract

Current Zika virus (ZIKV) outbreaks that spread in several areas of Africa, Southeast Asia, and in pacific islands is declared as a global health emergency by World Health Organization (WHO). It causes Zika fever and illness ranging from severe autoimmune to neurological complications in humans. To facilitate research on this virus, we have developed an integrative multi-omics platform; ZikaVR (http://bioinfo.imtech.res.in/manojk/zikavr/), dedicated to the ZIKV genomic, proteomic and therapeutic knowledge. It comprises of whole genome sequences, their respective functional information regarding proteins, genes, and structural content. Additionally, it also delivers sophisticated analysis such as whole-genome alignments, conservation and variation, CpG islands, codon context, usage bias and phylogenetic inferences at whole genome and proteome level with user-friendly visual environment. Further, glycosylation sites and molecular diagnostic primers were also analyzed. Most importantly, we also proposed potential therapeutically imperative constituents namely vaccine epitopes, siRNAs, miRNAs, sgRNAs and repurposing drug candidates.

Zika virus (ZIKV) is a flavivirus belonging to family Flaviviridae and is one of the major factors for current outbreak spreading over several areas of Africa, Southeast Asia, and in pacific islands. Zika infection was declared an emergency epidemic threat worldwide by World Health Organization (WHO) in early 2016 (http://www.who.int/en/). ZIKV is a mosquito-borne virus transmitted through monkeys and *Aedes* genus^1^ where humans are their occasional hosts. Majority of infections caused by virus are asymptomatic but cause slight illness called as Zika fever that leads to headache, rash, malaise and chills etc^2^. However, recent epidemiological studies suggest its association with neurological defects such as Guillain-Barre syndrome^3^,^4^, and microcephaly^5^,^6^. Besides the zoonotic and mother to child transmission of virus, Zika is even deemed as a sexually and transfusion-transmitted illness^7^,^8^. General life cycle events of ZIKV are depicted in Supplementary Figure S1.

The virus causing the disease was first isolated from serum sample of rhesus monkey^2^ and then in 1948 it was isolated from a group of *A. africanus* mosquitoes in Zika forest^2^. Subsequently, ZIKV infection was identified in African continents, i.e., Uganda (1948)^9^, Nigeria (1971)^10^, Sierra Leone (1972)^11^, Gabon (1975)^12^, southeastern part of Central African Republic (1979)^13^, French Polynesia (2013–14)^14^ and in Brazil (2015)^15^. Additionally, similar cases were also reported in Asian countries like Malaysia (1996)^16^, Yap state of Micronesia (2007)^17^, Pakistan^18^ and Cambodia in 2010^19^.

ZIKV contains single-stranded positive sense RNA genome of about ~11 kb^20^. It encloses one open reading frame that encodes a polypeptide of 3419 amino acids and 2 adjoining non-coding regions (NCR), i.e., 5′and 3′ NCR^20^. Polyprotein of virus is proteolytically processed into three major structural proteins namely capsid, precursor of membrane and envelope protein (E), seven non-structural proteins (NS1, NS2A, NS2B, NS3, NS4A, NS4B, NS5)^21^ and glycoprotein^22^,^23^. ZIKV infection symptoms are similar to other arboviral diseases like dengue. Diagnosis based on symptoms is unreliable for specific identification. Therefore, laboratory diagnosis is vital to obtain conclusive results^17^,^24^. Hence, appropriate selection of molecular diagnostics primers is significantly important for routine ZIKV or flavivirus identification.

To date there is no explicit antiviral drug treatment for combating its infection; only the symptoms can be mitigated^21^. Furthermore, the course of vaccine and drug development is extremely multifaceted, which may take several years for delivering specific anti-ZIKV vaccines^25^,^26^. Moreover, the tedious conventional vaccine development strategies make the situation worse^27^. Thus, *in silico* approaches are beneficial in revealing potential vaccine candidates^28^. Hence, an integrative approach comprising of proteome-scale screening and immunoinformatics is applied for predicting the putative yet promising vaccine candidates. The epitope-driven vaccine development approach has proved advantageous against several infections^29^,^30^,^31^,^32^ and recently an epitope-based vaccine called “RTS,S” (also known as Mosquirix^TM^)^33^,^34^ has effectively moved to phase-III trials utilizing an engineered T-cell epitope of the causative protozoan parasite (http://www.malariavaccine.org/). After successful clinical trials, it will be the first commercial vaccine against malaria^33^,^34^.

Alternatively, there are other strategies to develop effective therapeutic regimens. Like, RNA interference (RNAi) technology is extensively used in silencing of gene. Small interfering RNAs (siRNAs) are tested as new potential therapeutics^35^ against various pathogens and disorders^36^,^37^,^38^. They are often employed for focused anti-viral therapies^39^ against viruses including Hepatitis C virus (HCV) and Ebola^40^,^41^,^42^,^43^. Presently, over twenty siRNA-based therapeutics are in clinical trials^44^ including normal as well as chemically modified siRNAs (cmsiRNAs)^45^. These include SPC2996 for leukemia, EZN3042 for solid tumors and SPC3649 for HCV infection respectively^44^.

Additionally, microRNAs (miRNAs) are also found to play an important role in viral infections and activation of innate immune response^46^. Therefore, systematic genome wide screening of ZIKV genome for miRNAs may assist in designing anti-viral therapeutics including anti-miRs against ZIKV miRNAs. Further, predicting miRNA targets (in human and ZIKV) may help in understanding disease progression. Recently, in case of ZIKV, computational studies on predicting siRNAs^47^ as well as epitopes^48^ have been executed but no such database exists, which describes all the predicted siRNAs and epitopes in a well-defined and comprehensive manner. Lately, Clustered regularly interspaced short palindromic repeats (CRISPR)/CRISPR-associated proteins (Cas) approach has been developed for genome editing^49^,^50^,^51^. In this approach, small guide RNAs (sgRNAs) are utilized to alter genomes of various organisms from humans to viruses. Hence, it is also crucial to have complete list of sgRNAs for specific and efficient targeting of ZIKV through CRISPR/Cas technique.

In addition, for suggesting potential drugs that may combat Zika infection, therapeutic switching approach can be implemented. Over decades, this strategy of drug repositioning is extensively being exploited for allocating novel applications to existing drugs for different diseases^52^,^53^. Since, there is neither a drug available to treat Zika nor any drug has entered the drug discovery process. Therefore, computational approach leading to therapeutic switching could provide valuable insights in revealing potential drugs that may be effective against Zika infection.

However, to best of our knowledge no resource is available that is devoted to ZIKV comparative genomics, therapeutics and related analysis. Thus, to better understand different aspects, we have developed an integrated web-based multi-omics platform-**ZikaVR**. Mainly, therapeutically essential components like putative epitopes, siRNAs, miRNAs, sgRNAs (CRISPR/Cas9 targets), molecular diagnostic primers, related drug candidates and drugs information for therapeutic interventions against ZIKV are provided in the resource. Additionally, we have also developed graphical genome browser, “ZikaVR browser” for the collective representation of annotation and regulatory information.

## Utility and Discussion

**Figure 1:**
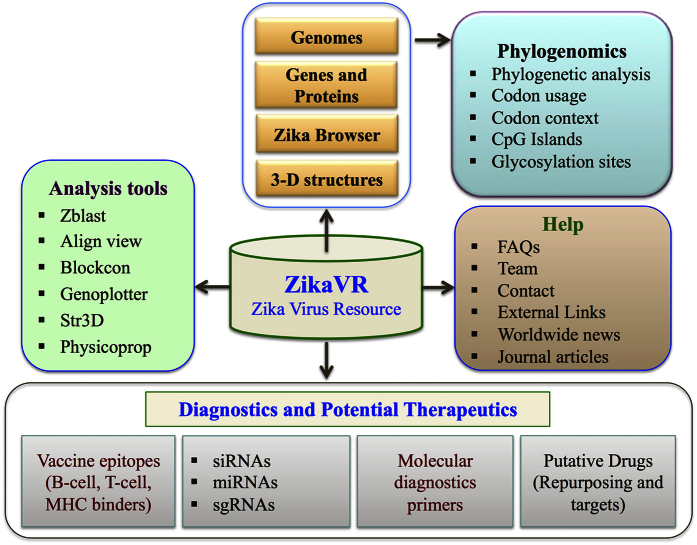
ZikaVR overview.

A systematic approach is applied for building ZikaVR wherein interesting findings have been exclusively assimilated in the resource. It is a well-structured and interactive platform, which supports high-performance genomic browser along with numerous comparative genomics analysis information and therapeutically important components. It is organized into various divisions like genomes, annotation browser, genes and proteins, epitope map, phylogenomics, molecular diagnostic primers, therapeutics that contains sub-divisions, i.e., vaccine epitopes, siRNAs, miRNAs, sgRNAs, and drug targets etc. Further, it also supports various tools for analysis and visualization of genomic content. It comprehends different tools like Zblast, Align viewer, Blockcon, Genoplotter, Str3D and Physicoprop (Fig. 1). This resource will certainly assist scientists and pharmaceutical agencies in conceiving experiments for enriched development of vaccine and drugs against ZIKV.

## ZikaVR genomes, proteomes and browser

**Figure 2:**
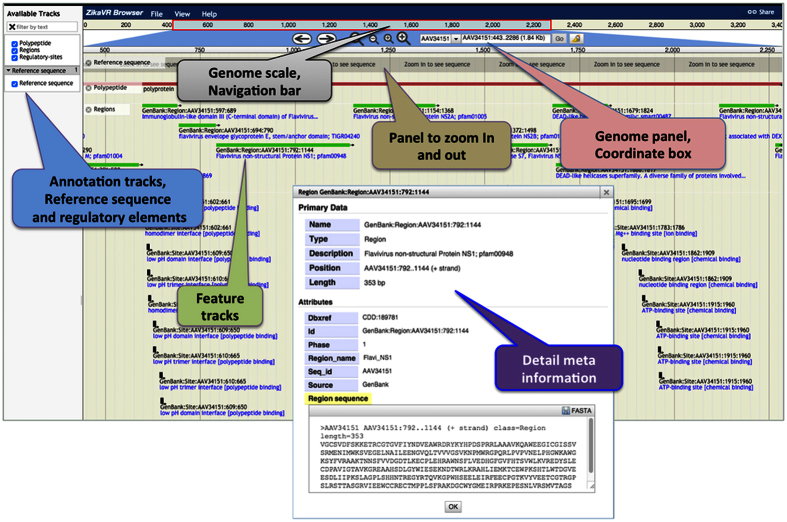
Overview of ZikaVR genome annotation browser. The upper panel shows the positional scale (ruler) to navigate through genomes along with Zika virus reference sequence. Distinct annotation features were shown in separate color blocks. Semantic navigation and zooming provide interactivity to browser.

All genomic information and annotation of ZIKV were compiled to provide highly sophisticated and informative user end interface. To navigate through the genomes and proteomes, we set up a ZikaVR browser, which facilitates dynamic graphical visualization of annotations (Fig. 2) powered by JBrowse as also implemented in ViralEpi v1.0^54^ and HPVbase^55^. Along with this, individual genome sequence analysis is also represented in static mode with circular representation of viral genomes (Supplementary Figure S2) and distinct analysis outcomes. Additionally, resource also facilitates an advance genome search page for easy retrieval of sequence data. User can search and categorize ZIKV genomes based on their status (complete or partial), geographical area (Africa, Asia etc.), country (Uganda, Thailand etc.), year and length. It supports flexible and smooth zooming, scrolling and browsing at different levels to display detailed information.

## Structural elucidation of Zika virus proteins

In order to extensively understand the ZIKV infection mechanism and underlying processes, fundamental requisite is the protein structure information. In pursuit of developing potential vaccines and drugs against Zika, promising targets have to be identified. For this purpose, Zika protein (Capsid, Envelope, Membrane glycoprotein, NS1, NS2A, NS2B, NS3, NS4A, NS4B and NS5) sequences were subjected to *in silico* structure prediction analysis. Recently, few structures of Zika proteins^56^,^57^,^58^,^59^,^60^ have been reported in the Protein Data Bank (PDB) (http://www.rcsb.org/pdb/home/home.do) and therefore the same structures were used as templates in our modeling procedure along with other structure templates. Overall, 840 tertiary structures of ZIKV proteins were modeled. List of PDB IDs used as template to model ZIKV proteins are provided in Supplementary Table S1. All the predicted structures for Zika proteins are provided in the resource with Jmol visualization facility and can be downloaded as PDB files. These protein structures will help in estimating the binding of drugs to potential drug targets^61^.

## Phylogenomics

We analyzed whole genome sequences of ZIKVs (85) and other viruses of genus *flavivirus* (10) to infer the evolutionary relationships and patterns. Here, we used comprehensive approach, which includes use of phylogenetic analysis, codon usage bias and context analysis, along with implication of conserved and variable region in diagnostics and therapeutics. The consolidated approach used in this study could provide better insight into evolutionary pattern and classification.

## Phylogenetic analysis

**Figure 3:**
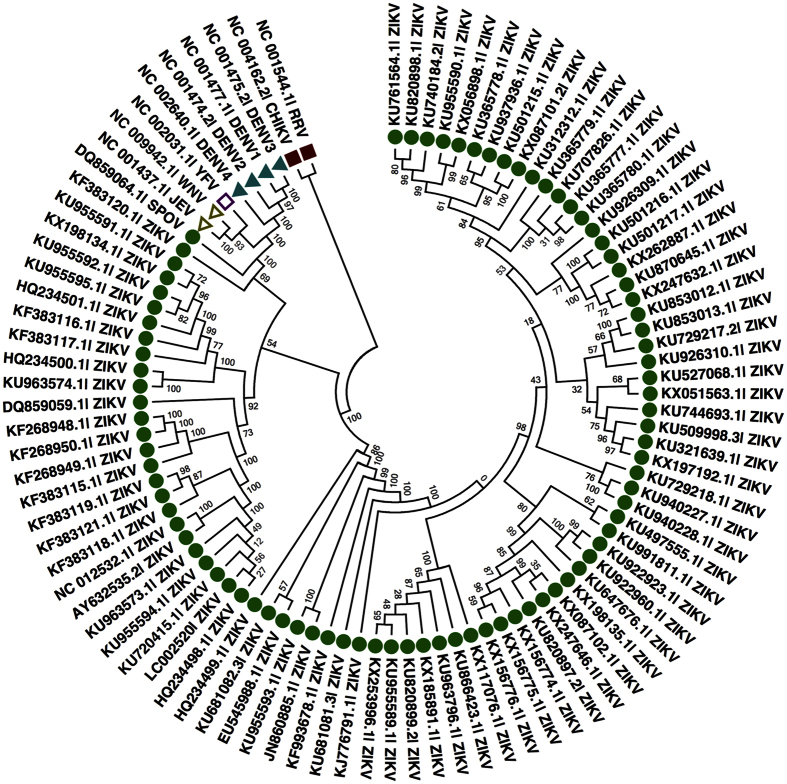
Circular tree representing phylogenetic relationships of 85 ZIKV genomes with other *flavivirus* (10) using maximum likelihood (ML) method; ZIKV; Zika virus, SPOV; Spondweni virus, JEV; Japanese encephalitis virus, WNV; West Nile virus, YFV; Yellow fever virus, DENV; Dengue virus, CHIKV; Chikungunya virus, RRV; Ross River virus.

**Figure 4:**
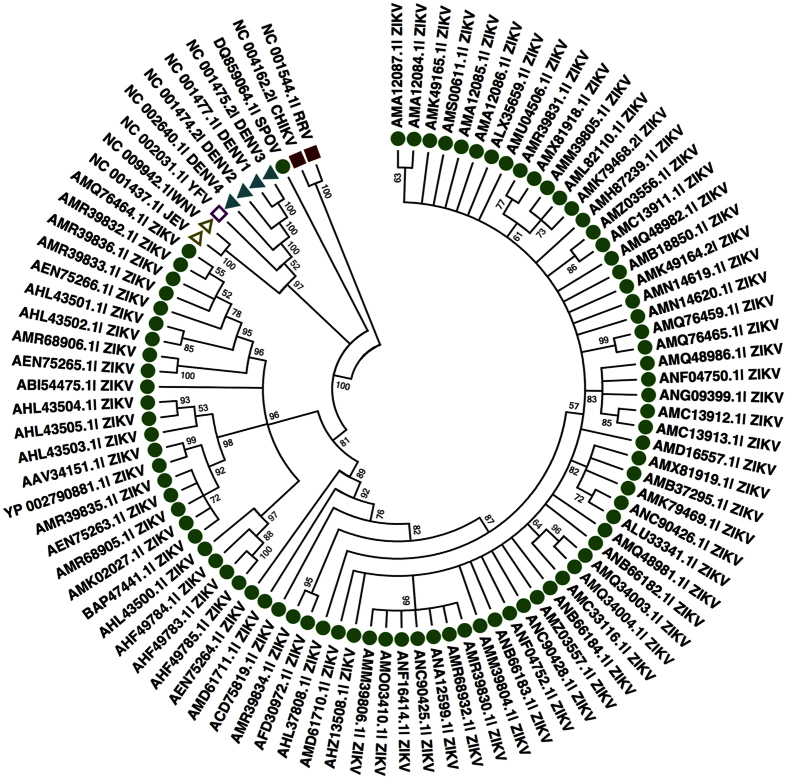
Tree representing phylogenetic relationships of 84 ZIKVs proteomes with other *flavivirus* (10) using maximum likelihood (ML) method. ZIKV; Zika virus, SPOV; Spondweni virus, JEV; Japanese encephalitis virus, WNV; West Nile virus, YFV; Yellow fever virus, DENV; Dengue virus, CHIKV; Chikungunya virus, RRV; Ross River virus.

All the 95 genomes (Fig. 3) and 94 proteomes (Fig. 4) were taken from five groups of viruses, i.e., Spondweni virus (SPOV), Dengue virus, Yellow fever virus, Japanese encephalitis virus, Semeliki forest virus groups. In Maximum likelihood (ML) analysis, ZIKVs belonged to Spondweni group and are arranged in different clades (Figs 3 and 4). They belong to various geographical regions like South America, North America, Asia, Uganda, Central African Republic, Senegal, etc. Similar pattern of phylogeny were reported in previous studies based on different gene regions^1^,^62^,^63^,^64^,^65^,^66^. SPOV shows close relation to ZIKV by both methods. Whereas, as expected outgroups from *togaviridae gp.* (CHIKV and RRV) showed distinctness from *flaviviridae gp*.

## Codon usage biasness and context

**Figure 5:**
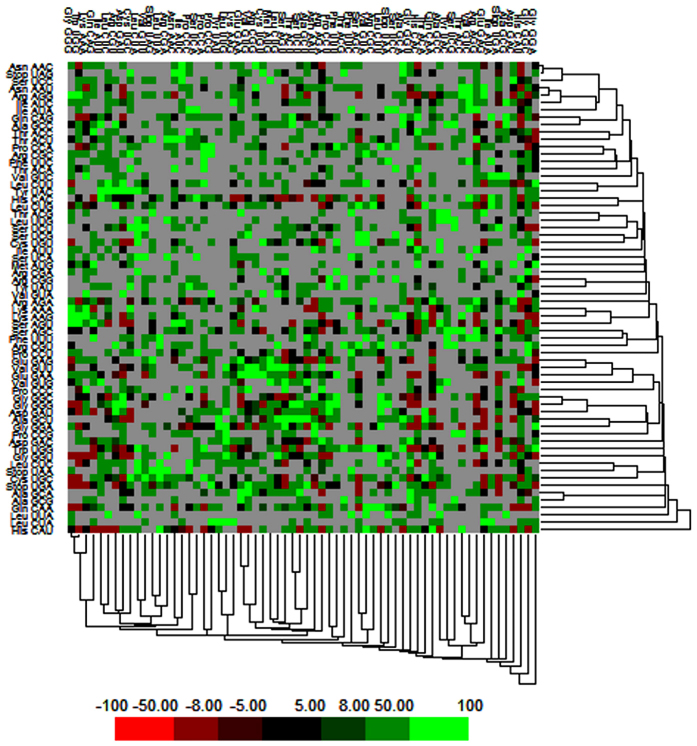
Matrix representing codon context analysis.

The pattern and usage frequencies of codons vary between and within genomes. It is affected by various factors mainly comprising gene length, nucleotide composition bias, G+C content, recombination events and rates, expression level etc. This biased usage of synonymous codons may be significantly useful to indicate and understand pattern of genome evolution among species. We have calculated codon context by utilizing Anaconda software. This software helps to calculate familiar residual values for involvement of each codon pair in genomes. This residual value indicates association between two codons of each context through chi square test. Average residual values were calculated for the total number of codon pairs. Each value in a cell of the frequency table was changed into a two-colored map as shown in Fig. 5. In the matrix, green (value more than +3) and red (value more than −3) color represents the preferred and rare codons, respectively.

**Figure 6:**
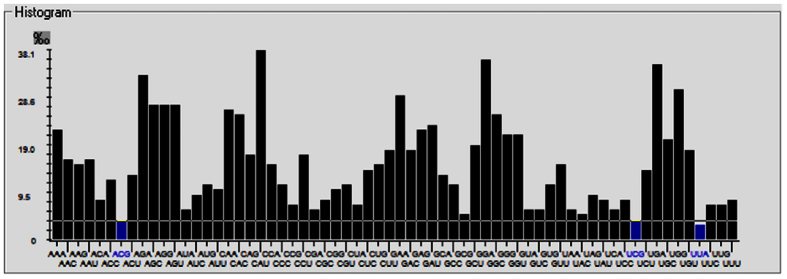
Codon usage graph depicting rare (blue) and preferred codons (black).

The cluster pattern in the matrix shows differences as well as commonalities of codon context between species. Similarly black color in the cells signifies that residual values fall within range of −3 to +3 and relates to codon context that does not correspond to biasness. We have also represented codon preference in the form of histogram in which blue color corresponds to rare and black color signifies preferred codons as shown in Fig. 6. This histogram shows that CAU, GGA, and UGA are the most preferred codons while ACG, UCG and UUA are amongst the rare codons present in NC_012532.1 strain of ZIKV. Detailed information of preferred and rare codons of other ZIKV strains is provided in ZikaVR resource. Our results provide useful insights on codon context and usage bias patterns that may facilitate better understanding of genome organization (Figs 5 and 6).

## Glycosylation patterns in ZIKV

In this study, we have predicted glycosylation sites (N-, O- and C-linked) in all the ZIKV strains. N-glycosylation is a type of post translation modification (PTM) which plays an important role in viruses such as proteolytic process, protein trafficking, virulence, immune evasion, virus assembly, receptor binding etc^67^.

For N-linked glycosylation (N-GlcNAc), we found Asn-X-Ser/Thr motif in envelope, NS2A, NS3, NS4B, and NS5 proteins among all strains. In NS3, N-GlcNAc sites were present at positions 158, 249 and 568, while in NS4B at 64, 216 and in NS5 at 214 in all strains isolated from different hosts (human, monkey, and mosquitoes). However, N-GlcNAc sites at position Asn-154 in envelope and 149 in NS2A sequences were restricted to strains from human host. In ZIKV and west nile virus strains, the deletion of the potential glycosylation site in envelope protein could be related to serial passages of the virus in mouse brain that has been previously reported^66^,^68^,^69^.

In addition, we have also detected O-linked glycosylation (O-GalNAc) which is responsible for various biological activities such as virus/bacteria-host interactions, ligand recognition, signal transduction etc^70^. The most common O-GalNAc positions in envelope protein are (7, 47, 48, 170, 173), membrane glycoprotein (6, 8, 56), NS1 (290, 293), NS2A (2, 3, 19), NS2B (1, 5), NS3 (34, 135, 225, 245), NS4A (36), NS4B (51), and NS5 (215). Furthermore, we also revealed C-linked glycosylated sites in membrane glycoprotein, NS2B, NS3 and NS5. In membrane glycoprotein sequence (AHL43503) glycosylation site was determined only at one position (115). The function of C-mannosylation is still not clear, however it may have crucial role in secretion and enzymatic activity^71^. NS2B and NS3 were shown to be glycosylated at 121 and 234 positions, respectively. Similarly, in case of NS5 protein, we found glycosylation at 702-residue position in some strains (isolated from sentinel rhesus monkey and *Aedes opok*). In recent years, the role of glycosylation responsible for viral infection has become one of the most emerging fields in drug designing^67^. Some studies interlink the effect of altered glycosylation sites with diseases (such as cancer) facilitating the development of biomarkers or therapeutic targets^72^,^73^.

## Molecular diagnostics

We compiled a list of all the oligonucleotide primer pairs used for the detection of ZIKV till date. All the information related to primers, i.e., their sequence, orientation, genomic positions with respect to the reference genome, and GenBank IDs of the strains that have been experimentally isolated using the respective primers is available on the website.

The primers obtained were tested for their specificity against all the 85 genomes of ZIKV as listed in Table 1 and Supplementary Table S2. Genomes to which both forward and reverse primers mapped completely are the ones considered to be amplified by that particular primer pair. In case of 6 ZIKV specific primer pairs obtained, the primer pair Unnamed1 and Unnamed2 showed exact complementarity against maximum genomes, i.e., 82 and has also previously shown to be able to detect all 37 strains of ZIKV used in the respective study^24^,^74^. Also, poor complementarity of this pair against 10 other related flaviviral genomes suggest that this primer pair may be used as a pan-ZIKV pair.

Additionally, six universal flaviviridae primer pairs (Supplementary Table S2) used for detection of ZIKV strains in literature were also analyzed. Out of six, one universal flaviviridae degenerate pair unifor and unirev was predicted to specifically amplify 69 out of 85 analyzed ZIKV genomes. This primer pair was also specific for 7 of the 10 related out groups, i.e., West Nile virus, Spondweni virus, Japanese encephalitis virus, Dengue virus 1, 2, 3, 4. Thus, in cases where a patient needs to be tested against a number of flaviviruses depicting similar symptoms, this specific primer pair can be used. Furthermore, 108 normal and 145 degenerate potential primer pairs were designed for 85 ZIKV genomes. Majority of these primers could detect more than 90% of the genomes. Selected primer pair for each genomic region is provided in Table 2. Gene names and numbers of designed primer pairs are listed in Supplementary Table S3. The complete list is provided on the web resource. The compendium of experimentally used primers as well as the ones designed using stringent conditions in this study can be used for ZIKV detection and thus maybe extremely useful for diagnosis.

### Potential Therapeutics

#### Putative vaccine candidates

In the study, attempts have been made to identify potential Zika epitopes comprising of B-cell epitopes, T-cell epitopes and promiscuous MHC binders. The 9mers have been generated to analyze the Zika genome wherein the peptides mapped to human proteome are eliminated. A total of 641 B-cell epitopes with their respective peptide sequence, Lbtope scores and B-cell confidence are stated at the website. The peptides (WGNGCGLFG and VDRGWGNGC) scored highest in B-cell epitope prediction and are proposed to be targeted. 1458 T-cell epitope based peptides have been identified in ZIKV and reported in ZikaVR. Overall 6725 MHC class I and 1631 MHC class II focusing epitopes are also predicted and displayed at the website. The information includes peptide sequences, MHC class I and II alleles and their counts. Further, to comprehend the impact of these binding peptides, the results from IFNepitope and IL4pred methods have also been integrated in the resource. The interferon-gamma inducing and interleukin-4 inducing peptides provide insights in understanding the downstream immune processes. In the analysis, 722 peptides were predicted to induce interferon-gamma on binding to MHC class II alleles and the used method for prediction is also clearly shown. While, 1169 peptides binding to MHC class II alleles are reported to stimulate interleukin-4.

Moreover, the study revealed a few experimentally characterized epitopes through B-cell, T-cell and MHC assays among these putative epitopes. The information corresponding to the Zika epitopes, their assays used for validation and numbers of reported evidences are provided in ZikaVR. The epitopes namely, TYQNKVVKVL, YFHRRDLRL and YMWLGARFL were found to be reported through B-cell, T-cell and MHC assays to activate majority of arms in the immune system. Further based on our *in-silico* high throughput analysis, we are recommending 32 potential vaccine epitope candidates (Table 3).

### RNA based therapeutics

#### Small interfering RNAs and microRNAs

We extracted 10776 putative siRNAs utilizing VIRsiRNApred and desiRm software with variable predicted efficacy ranging from 0 to 100 percent in inhibiting the target mRNAs. The immunomodulatory impact of these siRNAs as predicted by imRNA program demonstrates the roles of these siRNAs in further invoking the immune system. Further, 521 predicted siRNAs using VIRsiRNApred showed 70 percent or more silencing efficacy. Moreover, off targets of siRNAs in human genome are also provided along with predicted siRNAs. Representative set of efficient siRNAs is provided in Supplementary Table S4. The potential siRNAs obtained by desiRm software with higher efficacy score (i.e., >0.80) and their immunomodulatory roles were deduced. List of representative efficient siRNAs is specified in Supplementary Table S5. The server provides an interactive view where one can access detailed siRNA related information including its sequence, efficacy scores and immunomodulatory scores. User can check the conservation of the ZIKV siRNA against other viruses using “*siTarConserve*” tool in VIRsiRNApred web server^75^. Viruses have been targeted using siRNAs and have shown positive results including the flaviviruses as Dengue, West Nile virus, Japanese encephalitis virus etc^36^.

Additionally, 15 ZIKV pre-miRNAs were predicted from VMir; while mature 5p and 3p sequences were extracted from each pre-miRNA using Mature Bayes tool (30 mature ZIKV-miRNAs) (Table 4). Apart from the mature miRNA sequences and their location information in pre-miRNA and ZIKV genome, we have extracted GC content and free energy secondary structures of all mature miRNAs. The minimum free energy (MFE) secondary structures are provided on the web server along with other details. Further, orthologous miRNAs and potential targets were identified. Using TargetScan script and seed-align tool of VIRmiRNA, we have listed 202 orthologous miRNAs mainly from viruses like Epstein–Barr virus (EBV), Human herpesvirus 6B (HHV-6), White Spot Syndrome virus (WSSV) etc., *Drosophila melanogaster* and *Homo sapiens*. Seed of most of the orthologous miRNAs were found to be orthologous to the ZIKV-MR32-3p followed by ZIKV-MD77-5p and ZIKV-MD34-3p. Moreover, we have identified 688 experimentally validated targets. Most of the orthologous targets were reported for ebv-miR-bart7; which is orthologous to ZIKV-MR66.

#### Single guide RNAs (sgRNAs)

Based on our analysis, we have obtained 1898 sgRNAs in the complete genome of ZIKV. The output is represented in tabular form displaying sgRNA sequences, PAM, strand, i.e., sense/antisense (+/−), start and end position of this 23 residue sgRNA in the genome and its total GC%. This will surely help to identify CRISPR targets against ZIKV prior to experimental procedures and will save time.

#### Identification of potential drugs via therapeutic switching

For estimating the putative drugs that may prove promising for fighting Zika infection, the Zika genome was mapped to existing drugs in DrugBank for closely related viruses as depicted in the drug targets section under Therapeutics option. The selection criterion was based on its identity and coverage with ZIKV where a minimum threshold of 52% identity was set for filtering. Majority of known drug targets like genome polyprotein (DENV-2 and DENV-3) mapped to the Zika genome polyprotein with around 80% identity and have well-known small molecules/drugs against them in DrugBank. These small compounds acting as drugs include ribavirin monophosphate (DrugBank ID: DB01693), S-adenosyl-L-homocysteine (DrugBank ID: DB01752) and alpha-L-fucose (DrugBank ID: DB04473) (Table 5). The precise pharmacological action of most of these compounds against Zika infection is not yet evinced. Although, ribavirin monophosphate is known to possess anti-viral activities by either lethal mutagenesis or inhibiting inosine monophosphate dehydrogenase (IMPDH) leading to decline in intracellular GTP levels^76^. The decreased GTPs indeed interfere with the viral growth by limiting viral protein synthesis. On the other hand, S-adenosyl-L-homocysteine is believed to halt the maturation of viral mRNA thus displaying anti-viral property by selective inhibition of methyltranferases^77^. The drug repositioning analysis delivered interesting findings as the drugs with maximum identity and coverage with known drug targets in DrugBank (Table 5) were aligned to all the Zika genomes available at ZikaVR. This illustrates that the proposed drugs may target all the Zika strains but in varying degrees based upon their similarity with the known drug targets; thus exemplifying the power of drug repositioning. This strategy of drug repositioning has delivered promising drug candidates^52^,^53^ that upon validations and successful clinical trials may be effectively used against ZIKV.

### Analysis tools

ZikaVR also facilitates very useful analysis and visualization tools to explore genomic and structural information. These include (1) Zblast: to find similarity and align query sequence to the ZIKV genomes and genes. The output of this tool is similar to standard BLAST output along with tabular representation. (2) Align viewer: alignment visualization tool to interactively visualize, edit and manipulate multiple sequence alignment. (3) Blockcon: this tool allows user to select conserved region of a DNA and protein sequences from multiple sequence alignment to use in phylogenetic analysis. Here, we have implemented Gblocks program^78^ to provide easy-to-use server with maximum functionality. User can also download different results using download option. (4) Genoplotter: a dot plot analysis tool to compare two genome sequences in two-dimensional plot powered by Gepard V1.30 program^79^. (5) Str3D: tool to visualize 3-dimensional structure of proteins implemented using Jmol (www.jmol.org/) an open-source java viewer. (6) Physicoprop: this tool provides graphical view of important physico-chemical properties of epitopes or peptides^80^,^81^.

## Materials and Methods

### Genomic and proteomic data collection

ZIKV whole genome and proteome sequences were retrieved and collected from the NCBI and GenBank databases. Total of 333 sequences were obtained till May 2016, which were manually checked for the presence and absence of well-reported ORFs and categorized. After curation, overall 94 complete (9 with ambiguous nucleotides (Ns)) and 239 partial genomes were provided. A comprehensive advance search option is implemented on the server for easy retrieval and classification of sequence data. From the genomic data, following information were extracted, i.e., strain, isolate, isolation source, genome size, region, geographical area, host, and year. Nucleotide and protein sequences of all the full-length ZIKV genomes were investigated. Our analysis comprised of two phases: first phase was the full-length genomic analysis and in the second phase we analyzed each gene sequence at nucleotide (nt) and amino acid (aa) level.

### Structural elucidation of Zika proteins

We modeled 10 proteins of the ZIKV for the determination of their tertiary structures. Proteomes were divided into different proteins using protein boundaries. First, we identified templates for each protein by performing BLAST search against PDB database and selected the templates with e-value less than 0.01. Next, we selected top 10 templates if the number of hits was more than 10. We used MODELLER software^82^ to build the homology model for each protein. However, no templates were available for ‘NS2A’ and ‘NS4A’ proteins. For them, we first performed clustering of the respective sequences at 95% identity cutoff using CD-HIT software^83^ to select representative sequences of the individual proteins. For ‘NS2A’ we obtained 2 sequences and for ‘NS4A’ we obtained 1 sequence. We used online I-TASSER structure prediction server^84^,^61^ for the prediction of tertiary structure of representative protein sequences. Next, we used the first model of predicted I-TASSER structure as a template to model rest of the respective protein sequences.

### Multiple sequence alignment

In the whole genome study, all genomes were aligned using MEGA version 6.06^85^. Multiple sequence alignment of these sequences was conducted using the ClustalW program^86^ with default parameters to explore the conserved sites among distinct ZIKV genomes; represented by 80% or above conservation criteria.

### Phylogenetic analysis

Genomes (85) and proteomes (84) of ZIKVs along with 10 viruses of genus *flavivirus* were analyzed to deduce evolutionary relationship among them. Phylogenetic relationships were constructed with ML algorithm in MEGA 6.06^85^. Firstly, 95 viral genomes and 94 proteomes (1 genome non-functional) were aligned using ClustalW algorithm integrated in MEGA 6.06. Further, General Time Reversible (GTR) using a discrete Gamma distribution (+G) model was used for ML tree for genomes. Likewise, LG using discrete Gamma distribution (+G) was employed for ML tree building for proteomes. Moreover, statistical support was calculated using bootstrap analysis for both the trees using 1000 pseudo-replicates.

### Codon usage bias and context study

We compared and summarized various ways to analyze codon usage such as RSCU (relative synonymous codon usage) values, nucleotide contents, ENC (Effective number of codons) values^87^ etc. The number of times (row frequency) a codon is used for each amino acid is also utilized to analyze codon bias. Complete genomic sequences were analyzed using the CUSP (Create a codon usage table) program of EMBOSS (The European Molecular Biology Open Software Suite, Cambridge, UK). Additionally, we have also analyzed rare and preferred codon distribution and codon context among ZIKV strains using Anaconda program^88^.

### Glycosylation sites

We investigated capsid, envelope, membrane glycoproteins, NS1, NS2A, NS2B, NS3, NS4A, NS4B, NS5 protein sequences of ZIKV strains using NetCGlyc v1.0^89^, NetOGlyc v4.0^90^ and NetNGlyc v3.1^91^,^92^. These algorithms are based on neural networks to predict C-mannosylated, mucin type GalNAc O-linked and N-linked glycosylation sites respectively.

### Molecular diagnostic primers

Literature was thoroughly examined for the experimentally used primers for detection and diagnosis of ZIKV infections. These PCR primers were extracted and checked for specificity against about 85 ZIKV genomes. Additionally, potential candidate primers were also designed for these genomes using PrimerDesign-M tool^93^ with default parameters except following. The primers were designed for multiple fragments in a given region of interest based on the multiple sequence alignment where columns having more than 5% gaps were not considered. Flexible parameter for fragment overlap was selected. Complexity limit was taken to be 2 (i.e., one degenerate position allowed).

### Epitopes

The epitope identification focused on generating 9 mer overlapping peptides from 5 proteins namely, polyprotein, envelope protein, glycoprotein, NS3 and NS5 encoded by ZIKV genome. These peptides were further analyzed for their immune potential and were exclusively reduced to those 9mer-peptides specific to the virus consequently absent in human genome thus lowering the risk of self-tolerance. Further, human thousand proteomes were constructed by translating sequences from The 1000 Genomes Project into proteins^94^. Now, the viral peptides exhibiting 100% identity with the human thousand proteome were eliminated from the final analysis. After generating peptides with ZIKV-specific 9mer residues, the next objective was to narrow down the search to the peptides that may induce immune response and produce memory cells in human against the virus. For an advanced perspective on the immunomodulatory impact of these peptides, three major variants of epitopes, i.e., B-cell epitopes, T-cell epitopes and MHC alleles binding peptides were deliberated.

### B-cell epitopes

The linear and conformational B-cell epitopes in ZIKV were predicted from LBtope^95^ and CBTOPE^96^ methods respectively. LBtope is an efficient method built on huge dataset of experimentally validated B-cell epitopes and non-epitopes. For increasing the reliability of prediction, a cut-off of 60% was chosen for this prediction method. Another used method, i.e., CBTOPE has a distinctive feature for estimating the conformational B-cell epitopes from its primary structure where a threshold of −0.3 was shortlisted for prediction. The results from both the methods were integrated and are exhibited at the website.

### MHC allele binding peptides

In the present study, for predicting MHC class I binders in ZIKV, Propred1 tool has been utilized and top 4% have been selected^97^. This method works on a matrix-based program for predicting the binding peptides to MHC class I alleles. As these peptide binders for MHC molecules are known to activate cytotoxic T lymphocytes (CTL) therefore these are referred to as putative CTL epitopes, which were detected via CTLPred^98^. Likewise, MHC class II binders were predicted to estimate the potential T Helper (Th) epitopes in ZIKV using ProPred^99^. The top 3% of identified peptides have been proposed as promiscuous MHC class II binders.

### T-cell epitopes and immune response prediction

After the postulation of MHC binders that act as probable T-cell epitopes, CTL epitopes were also predicted discretely from CTLPred built on artificial neural network (ANN) and support vector machine modules. This tool directly focuses on antigen primary sequence and excludes the step for MHC class I binder’s prediction. For the detection of CTL epitopes in ZIKV, SVM-based module with default constraints have been implemented. All these predicted epitopes were also searched in The Immune Epitope Database (IEDB), the largest database on experimentally validated epitopes or antigenic regions^100^. The experimentally confirmed IEDB epitopes were mapped to Zika antigens for revealing the potential epitopes.

In addition, the interleukins released by MHC class II binders were also estimated using IFNepitope method at a default threshold of 0. The earlier predicted peptides can further stimulate Th1 cells (T-helper cell type I)^101^ guiding the release of interferon-gamma (IFN-γ). On similar lines, antigenic regions triggering Th2 (T-helper cell type II) cells for circulating cytokine, interleukin-4 (IL4) were predicted from IL4pred (default base of 0.2)^102^. These predicted epitopes and their subsequent impact on further release of immune regulators facilitates appropriate designing of vaccine candidates by broadly understanding the progression of Zika infection.

### Small interfering RNAs and microRNAs

Various databases are present in the literature for the viral siRNAs e.g. VIRsiRNAdb^36^ but no siRNA is so far designed for the ZIKV. We have employed VIRsiRNApred^75^ and DesiRm software^103^ for predicting the siRNAs against the ZIKV reference genome along with off-target information. Further, highly potent siRNAs (efficacy > 0.80) with their immunomodulatory impact predicted via imrna program^104^ were identified. Furthermore, we have used VMir algorithm^105^ for the detection of putative microRNA hairpins (pre-miRNAs). All predictions on VMir were carried out using the default parameters. Mature Bayes tool^106^ was used to identify mature miRNAs from the hairpin pre-miRNAs. Target predictions for the predicted ZIKV miRNAs were done using Tar-Find tool of VIRmiRNA^107^. Secondary structure of ZIKV miRNAs is also computed using RNAfold program of the Vienna Package^108^. Additionally, we have utilized the concept of orthology-based miRNAs and their targets prediction. Similar to TargetScan methodology, we have aligned seed sequences of ZIKV miRNAs to experimentally known viral as well as other cellular miRNAs using seed-align tool of VIRmiRNA.

### Single guide RNAs (sgRNAs) identification

For this, we have developed an in-house Perl script for the identification of all possible sgRNAs on the basis of Protospacer adjacent motif (PAM) in the ZIKV genome. This scans all “NGG” motifs in the genome of ZIKV on both the strands (forward and reverse) and then extracts 20 nucleotides upstream of the motif as putative sgRNA or CRISPR targets.

### Drug Repositioning

For this purpose, the Zika genome was mapped to existing drugs in DrugBank^109^ for related viruses. The ZIKV genome was subjected to repositioning for testified drugs in DrugBank that perhaps have approved toxicity and other safety regulations against closely related viruses. This approach of therapeutic switching reveals crucial findings and is judicious as it reduces the encountered costs during clinical trials. This analysis indicates that well-characterized drugs for related infections could probably be tested for fighting Zika as well. Thus, reducing complexity of determining effective drugs and rendering drug discovery process simpler.

### Development and implementation of ZikaVR

The ultimate challenge was to build exclusive portal integrating information from all the postulations constituted from above analysis as well as already available knowledge on ZIKV. An integrated resource has been designed that may assist scientific community concerned in developing therapeutics against ZIKV. This platform has been built on Linux operating system using Apache HTTP Server (version 2.2.17). Back-end of server is supported by MySQL (version 5.0.51b) for ensuring proper storage and management of data. The interface is created using HTML5, CSS3, PHP (version 5.2.14) and JavaScript (version 1.7) as previously implemented^55^,^43^,^110^,^111^, which complements its usage over a wide range of devices like laptops, mobiles and tablets. A number of in-house Perl and Python scripts were written for predicting putative epitopes, siRNAs, drug targets and potential drugs for ZIKV. A generalized workflow for ZikaVR is depicted in Fig. 1 offering an effortless comprehension to the developed compendium.

### Future developments

Advent of next generation sequencing (NGS) platforms facilitates to decipher specific disease single nucleotide variants (SNVs), mutations, viral integrations, epigenetic events etc. In future, we will develop and provide sophisticated NGS data analysis tools and pipeline to study these events. Additionally, as viral variations are influential in the pathogenicity, we will provide cataloging of existing known variations relevant to distinct diseases, which could provide comprehensive basis for personalized medicine. Additionally, other information can be extended; epitope structures and its visualization can also be provided. It may guide researchers in the development of effective therapeutic solutions and vaccines. We will continue to maintain steady operation and quarterly or half-yearly updation of ZikaVR.

## Conclusions

The intensification of Zika epidemic critically demands international collaborative efforts to reduce the risk of further spread in concerned regions and prevent the threat entering into non-affected countries. A lot of research focusing on ZIKV, its transmission mode, pattern of instigating infection and perceiving underlying mechanisms for inducing neurological abnormalities is the fundamental need of the hour. An interdisciplinary approach can address the issue of determining efficient vaccine candidates against ZIKV infection. Currently, there are limited *in silico* studies executed on ZIKV and Zika etiology whereas no such resource or compendium exists till date. In the developed resource, i.e., ZikaVR, majority of tools and methods used for the analysis are established by our own group. The preference of using these methods over other equally good software packages is the availability of standalone version for analyzing huge number of sequences and its comparable performance. ZikaVR provides a unique blend of interactive genomic annotation browser, comparative genomics and therapeutic analysis. Various components such as 3D structures, whole genome alignments, phylogenetic studies, genomic rearrangement and syntenic regions, codon usage and context are useful for comparative and evolutionary analysis especially important for diverge applications, i.e., epidemiological studies, taxonomy, comparative genomics, structural analysis etc. Further, molecular entity (i.e., primer) is critically essential for diagnostics. Based on our in-depth computational analysis, we are recommending and providing list of 32 potential vaccine epitope candidates. Similarly, siRNAs, miRNAs, sgRNAs and repositioned drug candidates are also advocated. We developed a user-friendly interface and dynamic resource with seamless functioning and sophisticated analysis tools. It is anticipated that ZikaVR will provide a valuable and comprehensive resource for genomic, evolutionary and therapeutic aspects of ZIKVs making it utilizable for wider research community. The predicted therapeutic targets in the study could be utilized for designing effective vaccines, and drugs to combat ZIKV.

## Additional Information

**How to cite this article**: Gupta, A. K. *et al.* ZikaVR: An Integrated Zika Virus Resource for Genomics, Proteomics, Phylogenetic and Therapeutic Analysis. *Sci. Rep.* **6**, 32713; doi: 10.1038/srep32713 (2016).

## Supplementary Material

Supplementary Information

## Figures and Tables

**Table 1:** ZIKV specific primers to amplify the genomic regions of the reference genome.

Primer	PMID	Sequence (5′-3′)	Genomic region (NC_012532.1)	Positions (NC_012532.1)	Mapped no. of genomes (85)
ZIKV 835	18680646	TTGGTCATGATACTGCTGATTGC	Membrane glycoprotein M	941 to 963	57
ZIKV 911c*		CCTTCCACAAAGTCCCTATTGC	Envelope protein E (3 m)	996 to 1017	
ZIKV 1086*	18680646	CCGCTGCCCAACACAAG	Envelope protein E (2 m)	1192 to 1208	57
ZIKV 1162c*		CCACTAACGTTCTTTTGCAGACAT	Envelope protein E (1 m)	1245 to 1268	
ZIKENVF	18674965	GCTGGDGCRGACACHGGRACT	Envelope protein E	1643 to 1663	30
ZIKENVR		RTCYACYGCCATYTGGRCTG	Envelope protein E	1989 to 2008	
ZIKAf	24507467	TGGAGATGAGTACATGTATG	Nonstructural protein NS3	6001 to 6020	14
ZIKAr		GGTAGATGTTGTCAAGAAG	Nonstructural protein NS4	6077 to 6095	
ZIKVF9027	22825831	CCTTGGATTCTTGAACGAGGA	RNA-dependent RNA polymerase NS5	9121 to 9141	61
ZIKVR9197c		AGAGCTTCATTCTCCAGATCAA	RNA-dependent RNA polymerase NS5	9291 to 9312	
Unnamed1	24148652	AARTACACATACCARAACAAAGTGGT	RNA-dependent RNA polymerase NS5	9365 to 9390	82
Unnamed2		TCCRCTCCCYCTYTGGTCTTG	RNA-dependent RNA polymerase NS5	9446 to 9466	

*Primers that are not perfectly complementary to the respective regions in the reference genome; m: mismatches.

**Table 2:** Designed ZIKV specific primers for each genomic region.

Genomic region	Primer Constructs		Start	Stop	Tm (C)
Anchored Capsid protein C	Frag2.F1.1	5′GATTGTCAATATGCTAAAAC	142	161	49.59
	Frag2.R1.1	5′TTTATTATTTCCATAGCCTC	332	351	49.36
Membrane glycoprotein precursor M	Frag2.F5.1	5′ACTTGGGTTGTGTACGGAAC	692	711	59.77
	Frag2.R5.1	5′TTGTCAAGGTAGGCTTCACC	1208	1227	58.94
Envelope protein E	Frag8.F4.1	5′TGTTTGGAGGAATGTCCTGG	2331	2350	59.02
	Frag8.R4.1	5′GGGAGTCAGGATGGTACTTG	2572	2591	59.21
Nonstructural protein NS1	Frag5.F2.1	5′AGAGGACCATCTCTGAGATC	3356	3375	57.08
	Frag5.R2.1	5′TCTCCTCCAGTGTTCATTTC	3731	3750	56.48
Nonstructural protein NS2A	Frag1.F2.1	5′GAGGACCATCTCTGAGATCAAC	3357	3378	59.63
	Frag1.R2.1	5′TGATCTTTGTGGTCATTCTCTTC	3607	3629	58.1
Nonstructural protein NS2B	Frag3.F1.1	5′GGAAAGAGTGTGGAYATGTAC	4346	4366	56.71
	Frag3.R5.1	5′CTATTGGGTTCATGCCACAG	4528	4547	58.51
Nonstructural protein NS3	Frag7.F5.1	5′CTCATAGCCTCGCTCTATCG	6107	6126	58.64
	Frag7.R5.1	5′CTCTCTGTCATGTGTCCTGG	6491	6510	59.03
Nonstructural protein NS4A	Frag2.F1.1	5′GAAGATGGTGCTTTGATGGC	6276	6295	59.03
	Frag2.R1.1	5′TCTCTGTCATGTGTCCTGGC	6490	6509	60.87
Nonstructural protein NS4B	Frag2.F1.1	5′TTGGAGTCCCGCTGCTAATG	7173	7192	61.94
	Frag2.R1.1	5′TCWATTGTCATTGTGTCAATGTCAG	7365	7389	59.48
RNA-dependent RNA polymerase NS5	Frag6.F1.1	5′ACACATACCAAAACAAAGTG	9369	9388	53.74
	Frag6.R1.1	5′CAATCAGTTCATCTTGGTGG	9847	9866	55.86

**Table 3:** List of recommended 32 potential vaccine epitope candidates.

ZikaVR ID	Epitope	LB Score	B Cell Confidence	MHC class II Alleles	MHC class II Alleles (Count)	IFN gama inducing score
ZIKA000466	LGGFGSLGL	0.34	61.49	DRB1_0102, 0402, 0404, 0405, 0410, 0423, 0701, 0703, 1501, 1502, 1506	11	0.64
ZIKA000541	VVVLGSQEG	0.74	74.79	DRB1_0101, 0102, 0401, 0402, 0404, 0405, 0408, 0410, 0421, 0423, 0426, 1301, 1304, 1327, 1328, DRB5_0101, 0105	17	0.50
ZIKA000542	VVLGSQEGA	0.63	71.01	DRB1_0301, 0305, 0306, 0307, 0308, 0311, 1107	7	0.33
ZIKA000701	VRGAKRMAV	0.37	62.49	DRB1_0102, 0301, 0305, 0306, 0307, 0308, 0309, 0311, 0701, 0703, 0802, 0804, 0806, 0813, 0817, 1101, 1102, 1104, 1106, 1107, 1114, 1120, 1121, 1128, 1301, 1302, 1304, 1305, 1307, 1311, 1321, 1322, 1323, 1327, 1328, 1501, 1502, 1506	38	0.54
ZIKA000720	VGGVFNSLG	0.51	67.07	DRB1_0404, 0405, 0410, 0423	4	0.13
ZIKA001120	WYGMEIRPR	0.38	62.83	DRB1_0802, 1101, 1102, 1114, 1120, 1121, 1128, 1301, 1302, 1305, 1307, 1322, 1323, 1327, 1328, DRB5_0101, 0105	17	0.86
ZIKA001121	YGMEIRPRK	0.45	64.84	DRB1_0305, 0309	2	0.66
ZIKA001410	YVVSGKSVD	0.53	67.67	DRB1_0301, 0305, 0309, 0801, 1321	5	0.17
ZIKA002295	MDIDLRPAS	0.60	69.95	DRB1_0301, 0305, 0306, 0307, 0308, 0309, 0311, 1107	8	0.19
ZIKA002366	YSQLTPLTL	0.48	66.16	DRB1_0101, 0703, 1101, 1128, 1305, 1321, DRB5_0101, 0105	8	0.29
ZIKA002714	LQRRHGGGL	0.55	68.2	DRB1_0801, 0802, 0804, 0806, 1506	5	0.84
ZIKA002897	WLWKELGKR	0.41	63.57	DRB1_0801, 0802	2	0.23
ZIKA002899	WKELGKRKR	0.50	66.76	DRB1_1114, 1120, 1128, 1302, 1305, 1307, 1323, DRB5_0101, 0105	9	0.78
ZIKA002992	WYMWLGARF	0.43	64.22	DRB1_1114, 1120, 1128, 1302, 1305, 1323	6	0.40
ZIKA002993	YMWLGARFL	0.99	83.31	DRB1_0101, 0102, 0701, 0703, 1114, 1120, 1302, 1307, 1323, DRB5_0101, 0105	11	0.32
ZIKA004499	IIGKRIERI	0.34	61.33	DRB1_0804, 0813	2	0.03
ZIKA004519	IKKRLRTLI	0.84	77.97	DRB1_0804, 0813, 1506	3	0.23
ZIKA004856	LERLQRRYG	0.41	63.6	DRB1_1102, 1114, 1120, 1121, 1301, 1302, 1304, 1322, 1323, 1327, 1328	11	0.39
ZIKA004986	LQRRYGGGL	0.84	78.13	DRB1_0801, 0802, 0804, 0806, 1506	5	0.97
ZIKA005005	LRRPEKVTN	0.56	68.79	DRB1_0806, 1304	2	0.50
ZIKA005011	LRWLVERGY	0.35	61.77	DRB1_1301, 1327, 1328, DRB5_0101, 0105	5	0.45
ZIKA005057	LWLLRRPEK	0.65	71.76	DRB1_1101, 1104, 1106, 1107, 1311	5	0.26
ZIKA005101	MERLQRRYG	0.47	65.78	DRB1_1102, 1114, 1120, 1121, 1301, 1302, 1304, 1322, 1323, 1327, 1328	11	0.02
ZIKA006392	WLLRRPEKV	0.80	76.57	DRB1_0801, 0802, 0813, 0817	4	0.58
ZIKA007151	FGRLVVLVG	0.99	83.56	DRB1_0101, 0309, 0401, 0405, 0408, 0410, 0421, 0426, 0801, 0802, 0813, 0817, 1101, 1104, 1106, 1114, 1120, 1128, 1301, 1302, 1305, 1307, 1311, 1321, 1323, 1327, 1328	27	0.55
ZIKA007187	FREQQGWGS	0.70	73.19	DRB1_0101, 1114, 1120, 1302, 1323	5	0.16
ZIKA007877	LAVLMRAEA	0.98	82.8	DRB1_0802, 0804, 1101, 1102, 1104, 1106, 1121, 1307, 1311, 1322	10	0.34
ZIKA007888	LDIDLRPAS	0.37	62.27	DRB1_0301, 0305, 0306, 0307, 0308, 0309, 0311, 1107	8	0.14
ZIKA008094	LYNAPKRLK	0.37	62.48	DRB1_0804, 1102, 1104, 1106, 1114, 1120, 1121, 1301, 1302, 1304, 1307, 1311, 1322, 1323, 1327, 1328, DRB5_0101, 0105	18	0.08
ZIKA008154	MLGCYSQLT	0.38	62.76	DRB1_0402	1	0.43
ZIKA008192	MTRKLLGST	0.35	61.59	DRB1_0806	1	0.08
ZIKA009088	VEFREAHAK	0.32	60.64	DRB1_0402, 0802, 0804	3	0.67

**Table 4:** List of predicted and analyzed 30 mature ZIKV-miRNAs.

ZIKVmiR ID	miRNA name	Mature sequence	VMiR score	Orientation	No. Orthologs miRNAs	No. of Predicted targets
ZIKV-MR110-5p	MR110-5p	UUUCCCCACACCGGCCGCCGAA	153.2	Reverse	2	NA
ZIKV-MR110-3p	MR110-3p	UGGCGGCCAGCGUGGUGGAAAC	153.2	Reverse	5	NA
ZIKV-MD19-5p	MD19-5p	AAGGAGCCGUUCACACGGCUCU	175.4	Direct	3	NA
ZIKV-MD19-3p	MD19-3p	UCUCGCUGGAGCUCUAGAGGCU	175.4	Direct	1	NA
ZIKV-MD100-5p	MD100-5p	UUUCCACCACGCUGGCCGCCAG	169.8	Direct	6	NA
ZIKV-MD100-3p	MD100-3p	CGGCGGCCGGUGUGGGGAAAUC	169.8	Direct	4	NA
ZIKV-MR16-5p	MR16-5p	CUGAAAAGUCAAGGCCUGUCCU	154.3	Reverse	5	NA
ZIKV-MR16-3p	MR16-3p	CUUCCGCUCUUGGUGAAUUAGG	154.3	Reverse	10	NA
ZIKV-MR61-5p	MR61-5p	AUGGCACAUCAAAUCAACGAUU	138.8	Reverse	5	1
ZIKV-MR61-3p	MR61-3p	UGACGUUGACUGCUGUUGUCAU	138.8	Reverse	4	NA
ZIKV-MD26-5p	MD26-5p	AAGGGCAUUCACCAGAUUUUUG	134.0	Direct	4	NA
ZIKV-MD26-3p	MD26-3p	AAAUCACUGUUUGGAGGAAUGU	134.0	Direct	3	NA
ZIKV-MR66-5p	MR66-5p	AGGCAUCUCCUAGAGUCUAUGA	133.7	Reverse	3	224
ZIKV-MR66-3p	MR66-3p	GUCUAUGACCCGGUCAGCCUUG	133.7	Reverse	3	224
ZIKV-MR1-5p	MR1-5p	CGAUGCUGGUGUCUGCGCCACG	133.1	Reverse	3	4
ZIKV-MR1-3p	MR1-3p	CGUCUCUUCCUCUCUUUCCUAG	133.1	Reverse	4	NA
ZIKV-MR43-5p	MR43-5p	UCAGUGCGUGGCACGGCCAUUG	132.0	Reverse	NA	NA
ZIKV-MR43-3p	MR43-3p	CCAUUGCUCGAAUUGCCAACCA	132.0	Reverse	3	NA
ZIKV-MD45-5p	MD45-5p	CCCUGAAAGGGAAAGGUAGUGU	131.5	Direct	4	192
ZIKV-MD45-3p	MD45-3p	AAAGGUAGUGUGAAGAAGAACC	131.5	Direct	2	NA
ZIKV-MD77-5p	MD77-5p	GGGACGGGAGAGACUCUGGGAG	119.9	Direct	24	1
ZIKV-MD77-3p	MD77-3p	CGUCUGAAUCAGAUGUCGGCCC	119.9	Direct	2	10
ZIKV-MR32-5p	MR32-5p	AUUCCCUACAGCACCAUUCCUC	118.2	Reverse	4	20
ZIKV-MR32-3p	MR32-3p	UGAUCUCAGAGAUGGUCCUCUA	118.2	Reverse	70	12
ZIKV-MD65-5p	MD65-5p	AUGCGGCCCUGAAGUCGUUCAA	117.3	Direct	2	NA
ZIKV-MD65-3p	MD65-3p	CUUUGGGAGUAAUGGAGGCCCU	117.3	Direct	6	NA
ZIKV-MR57-5p	MR57-5p	GGCUCCUUUUGUAACGUGCCAC	108.2	Reverse	2	NA
ZIKV-MR57-3p	MR57-3p	CGUGCCACAUGGUGUGGAAGAC	108.2	Reverse	2	NA
ZIKV-MD34-5p	MD34-5p	AGAGGACCAUCUCUGAGAUCAA	102.8	Direct	5	NA
ZIKV-MD34-3p	MD34-3p	AAGGGUCAUUGAGGAAUGGUGC	102.8	Direct	11	NA

**Table 5:** The proposed drugs for Zika virus via drug repositioning.

S. No	Drug name	Drug Bank ID	Known Drug Targets	Zika Protein IDs
1	Alpha-L-Fucose	DB04473	Genome polyprotein (Dengue virus type 2 and type 3)	YP_002790881.1, ALU33341.1, AMA12084.1, AMA12085.1, ALX35659.1, AHZ13508.1, AHL43504.1, AHL43503.1, AHL43502.1 AHL43501.1, AHL43500.1, AHF49785.1, AHF49784.1, AHF49783, ACD75819, ABI54475.1, AAV34151.1, BAP47441.1, AMA12087.1, AMA12086.1
2	Ribavirin Monophosphate	DB01693	Genome polyprotein (Dengue virus type 2 and type 3)	YP_002790881.1, ALU33341.1, AMA12084.1, AMA12085.1, ALX35659.1, AHZ13508.1, AHL43504.1, AHL43503.1, AHL43502.1, AHL43501.1, AHL43500.1, AHF49785.1, AHF49784.1, AHF49783, ACD75819, ABI54475.1, AAV34151.1, BAP47441.1, AMA12087.1, AMA12086.1
3	S-Adenosyl-L-Homocysteine	DB01752	Genome polyprotein (Dengue virus type 2 and type 3)	YP_002790881.1, ALU33341.1, AMA12084.1, AMA12085.1, ALX35659.1, AHZ13508.1, AHL43504.1, AHL43503.1, AHL43502.1, AHL43501.1, AHL43500.1, AHF49785.1, AHF49784.1, AHF49783, ACD75819, ABI54475.1, AAV34151.1, BAP47441.1, AMA12087.1, AMA12086.1
