# Supplementary material for: ZikaVR: An Integrated Zika Virus Resource for Genomics, Proteomics, Phylogenetic and Therapeutic Analysis
Source: Sci Rep. 2016 Sep 16;6:32713. doi: 10.1038/srep32713 (PMC5025660; doi:10.1038/srep32713)
Supplement: Supplementary Information [file srep32713-s1.pdf]

ZikaVR: An Integrated Zika Virus Resource for Genomics, Proteomics, Phylogenetic and Therapeutic Analysis

Amit Kumar Gupta[#], Karambir Kaur[#], Akanksha Rajput[#], Sandeep Kumar Dhanda[#], Manika Sehgal[#], Md. Shoaib Khan[#], Isha Monga[#], Showkat Ahmad Dar[#], Sandeep Singh, Gandharva Nagpal, Salman Sadullah Usmani, Anamika Thakur, Gazaldeep Kaur, Shivangi Sharma, Aman Bhardwaj, Abid Qureshi, Gajendra Pal Singh Raghava and Manoj Kumar*

Bioinformatics Centre, Institute of Microbial Technology, Council of Scientific and Industrial Research (CSIR), Sector 39A, Chandigarh-160036, India

[#]Authors contributed equally
*To whom correspondence should be addressed. Tel, 91-172-6665453; Fax, 91-172-12 2690585; 91-172-2690632; Email, manojk@imtech.res.in

Amit Kumar Gupta, Email: amitg@imtech.res.in
Karambir Kaur, Email: karman@imtech.res.in
Akanksha Rajput, Email: akanksha@imtech.res.in
Sandeep Kumar Dhanda, Email: sdhanda@imtech.res.in
Manika Sehgal, Email: manika.sehgal@imtech.res.in
Md. Shoaib Khan, Email: shoaibkhan@imtech.res.in
Isha Monga, Email: mongaisha@imtech.res.in
Showkat Ahmad Dar, Email: showkat@imtech.res.in
Sandeep Singh, Email: sandysingh@imtech.res.in
Gandharva Nagpal, Email: gnagpal@imtech.res.in
Salman Sadullah Usmani, Email: salmanusmani@imtech.res.in
Anamika Thakur, Email: anamikathakur@imtech.res.in
Gazaldeep Kaur, Email: gazaldeep@imtech.res.in
Shivangi Sharma, Email: shivangi@imtech.res.in
Aman Bhardwaj, Email: aman.bhardwaj@imtech.res.in
Abid Qureshi, Email: qureshisaab@imtech.res.in
Gajendra Pal Singh Raghava, Email: raghava@imtech.res.in
Manoj Kumar, Email: manojk@imtech.res.in

Supporting Information

Figure S1. **Zika virus life cycle and its circulation in secondary host namely monkeys and humans**. Mosquitoes (*Aedes* genus) act as a vector for Zika virus. Human babies are believed to develop microcephaly if the parents are infected with the Zika virus especially mother. (1) Mosquito bites the epidermal cells of the host and transfers virus particles into them. (2) Host cell bear the receptors for the attachment of E-Protein of Zika virus. (3) Internalization of the virus particle mostly by apoptotic mimicry as endosome. (4) Viral genetic material (+ sense ssRNA) is released into the cytoplasm. (5) Viral genome consists of the structural and non-structural proteins shown in different hypothetical color-coding. (6) Positive strand RNA is translated into single polyprotein on the rough endoplasmic reticulum (RER). (7) Replication occurs near endoplasmic reticulum (ER) in which double stranded RNA is formed to give rise to mRNA and new ssRNA (+). (8) Immature viral particle assembly occurs at the ER and the viral-vesicles bud off to fuse into Golgi apparatus. (9) Viral maturation occurs inside the Golgi complex. (10) Viral particles are released by exocytosis to outside of cell with pr protein cleaved off its M-protein. (11) Virons are transported via blood to different body parts and can spread to other hosts via mosquito.

Figure S2. Circular genome representation of Zika virus.

Table S1. List of PDB ids used to model zika virus protein structures.

Table S2: Universal flaviviridae primer pairs and the genomic regions, which these primers can amplify from the reference genome.

Table S3. List of gene name and number of designed primer pairs.

Table S4. Predicted highly potential siRNAs against Zika virus using VIRsiRNApred web server.

Table S5. Predicted potential siRNA suppressors for Zika virus using DesiRm web server.

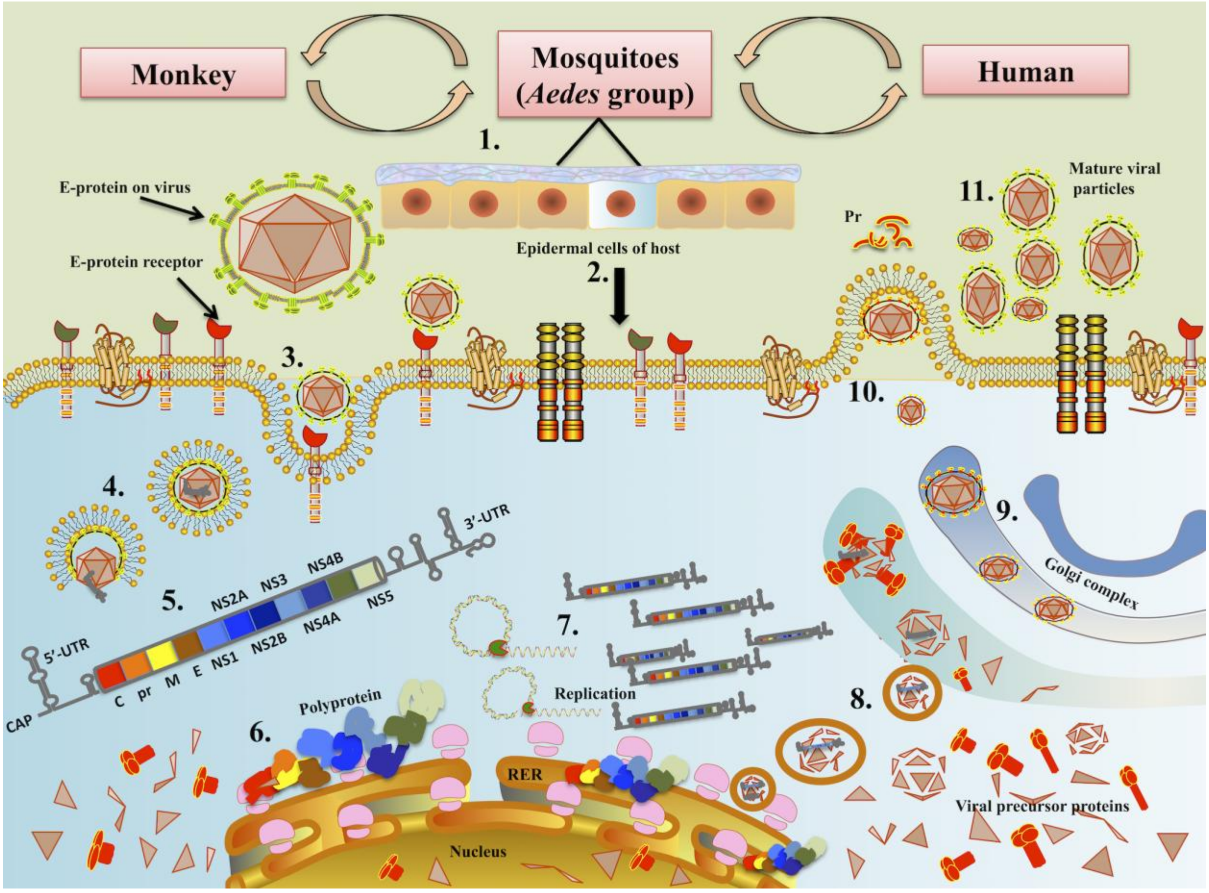

Figure S1. **Zika virus life cycle and its circulation in secondary host namely monkeys and humans**. Mosquitoes (*Aedes* genus) act as a vector for Zika virus. Human babies are believed to develop microcephaly if the parents are infected with the Zika virus especially mother. (1) Mosquito bites the epidermal cells of the host and transfers virus particles into them. (2) Host cell bear the receptors for the attachment of E-Protein of Zika virus. (3) Internalization of the virus particle mostly by apoptotic mimicry as endosome. (4) Viral genetic material (+ sense ssRNA) is released into the cytoplasm. (5) Viral genome consists of the structural and non-structural proteins shown in different hypothetical color-coding. (6) Positive strand RNA is translated into single polyprotein on the rough endoplasmic reticulum (RER). (7) Replication occurs near endoplasmic reticulum (ER) in which double stranded RNA is formed to give rise to mRNA and new ssRNA (+). (8) Immature viral particle assembly occurs at the ER and the viral-vesicles bud off to fuse into Golgi apparatus. (9) Viral maturation occurs inside the Golgi complex. (10) Viral particles are released by exocytosis to outside of cell with pr protein cleaved off its M-protein. (11) Virons are transported via blood to different body parts and can spread to other hosts via mosquito.

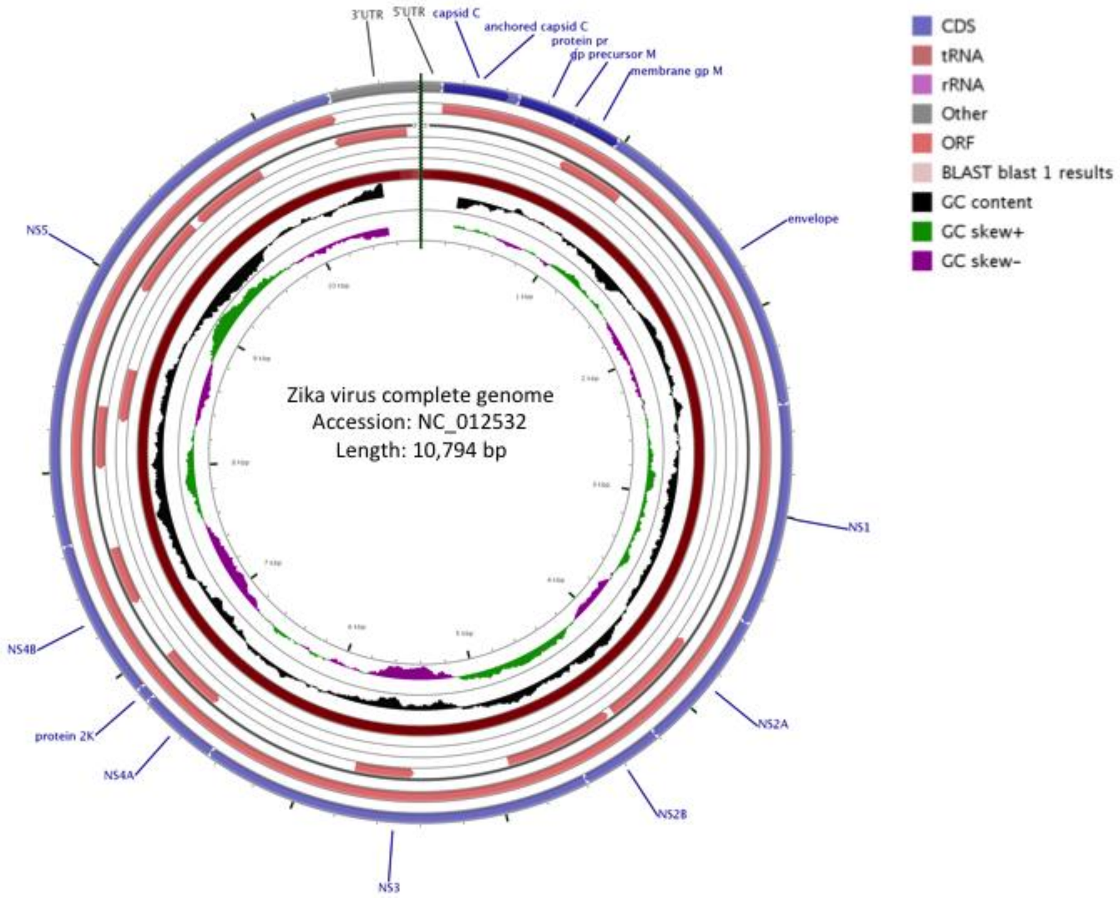

Figure S2. Circular genome representations of Zika virus

Table S1. List of PDB ids used to model zika virus protein structures.

Protein name	Templates (PDB IDs)
Capsid	1R6R, 1SFK
Glycoprotein	4B03, 5IZ7*, 5IRE*, 3C5X, 3IYA, 3C6R, 3J42, 3IXY, 3C6D
Envelope	5IZ7*, 5IRE*, 5JHM*, 5JHL*, 3J6U, 3J6T, 3J6S, 4CCT, 4B03, 4C2I
NS1	4O6B, 4TPL, 4O6D, 4O6C, 5IY3*, 4OIG, 4OII, 4OIE
NS2A	NA
NS2B	2YOL, 2FP7, 2IJO, 2GGV, 3E90, 2WV9, 2M9Q, 2M9P, 2M9M
NS3	5JMT*, 2WV9, 2WZQ, 2WHX, 2VBC, 2BMF, 2BHR, 2JLS, 2JLZ, 2JLX
NS4A	NA
NS4B	4K6M, 2PXC, 2PXA, 2PX8, 2PX5, 2PX4, 2PX2, 3LKZ, 2OY0, 4CTK
NS5	2HFZ, 4K6M, 4MTP, 5DTO, 4HDH, 4HDG, 4V0Q, 4V0R, 2HCS, 2HCN

*PDB IDs for experimentally determined zika protein structures

NA: Not available, (Strucutre for representative protein sequence is determined using I-TASSER then used as template for respective other protein sequences)

Table S2: Universal flaviviridae primer pairs and the genomic regions, which these primers can amplify from the reference genome.

Primer	Sequence	Genomic region (NC_012532.1)	Position (NC_012532.1)	Mapped (38)
EMF1	TGGATGACSACKGARGAYATG	RNA-dependent RNA polymerase NS5	10067 to 10087	0
VD8*	GGGTCTCCTCTAACCTCTAG	NA	10649 to 10668 (1 mismatch)	
Flav100F*	AAYTCIACICAIGARATGTAY	Envelope protein E	1478 to 1498 (10 mismatches)	0
Flav200R*	CCIARCCACATRWACCA	RNA-dependent RNA polymerase NS5	9080 to 9096 (2 mismatches)	
FU1*	TACAACATGATGGGAAAGAGAGAGAA	RNA-dependent RNA polymerase NS5	9011 to 9036 (2 mismatches)	0
cFD3*	AGCATGTCTTCCGTGGTCATCCA	RNA-dependent RNA polymerase NS5	10067 to 10089 (2 mismatches)	
PF1S5	TGYRTBTAYAACATGATGGG	RNA-dependent RNA polymerase NS5	9005 to 9024	0
PF2R-bis*	GTGTCCCAICCNGCNGTRTC	RNA-dependent RNA polymerase NS5	9257 to 9276 (1 mismatches)	
Unifor	TGGGGNAAYSRNTGYGGNYTNTTYGG	Envelope protein E	1277 to 1302	27
Unirev*	CCNCCHRNNGANCCRAARTCCCA	Envelope protein E	2249 to 2271 (1 mismatch)	
Mounifor2	GGRDRMDTBKWSAYVTGYGCNAWRTT	Envelope protein E	1307 to 1332	1
Mounirev2*	CCNATNSWRCTHCCHKHYYTRWRCCA	Nonstructural protein NS2A	4035 to 4060 (5 mismatches)	

*Primers that are not perfectly complementary to the respective regions in reference genome

Table S3. List of gene name and number of designed primer pairs.

Gene name	No. of designed primer pairs
Capsid	13
Glycoprotein	10
Envelope	35
NS1	25
NS2A	20
NS2B	20
NS3	30
NS4A	30
NS4B	15
NS5	55

Table S4. Predicted highly potential siRNAs against Zika virus using VIRsiRNApred web server.

Sense	Antisense	Target Gene	Start position	End position	Inhibition
GAACCAGCCAGAATTGCAT	ATGCAATTCTGGCTGGTTC	NS4A	6752	6770	96.27
GCTCATGGTGTGGAATAGA	TCTATTCCACACCATGAGC	NS5	10087	10105	92.73
ATGAGGGAGTGGAACCAGA	TCTGGTTCCACTCCCTCAT	Protein pr	642	660	91.55
CAGCCAGAATTGCATGTGT	ACACATGCAATTCTGGCTG	NS4A	6756	6774	91.55
GAGTGGGAGTCATGCAAGA	TCTTGCATGACTCCCACTC	NS3	4710	4728	90.09
GAATGGTGCTGTAGGGAAT	ATTCCCTACAGCACCATTC	NS1	3404	3422	88.12
CATGGTGCGCAGGATCATA	TATGATCCTGCGCACCATG	NS5	10264	10282	87.41
AGGGAGTGGAACCAGATGA	TCATCTGGTTCCACTCCCT	Protein pr	645	663	87.13
GAAGCCATTGACAACCTCG	CGAGGTTGTCAATGGCTTC	NS4A	6518	6536	87.02
TCATGGTGTGGAATAGAGT	ACTCTATTCCACACCATGA	NS5	10089	10107	86.93
GGTGAAGCCTACCTTGACA	TGTCAAGGTAGGCTTCACC	Envelope protein E	1208	1226	86.49
GAAAGAGTGTGGACATGTA	TACATGTCCACACTCTTTC	NS2B	4347	4365	85.98
GCAGGAGCTTCCCTTATCT	AGATAAGGGAAGCTCCTGC	NS4B	7598	7616	85.94
GTAGGCTTGCTAATTGTCA	TGACAATTAGCAAGCCTAC	NS2B	4313	4331	85.92
CGTTGTGGATGGAATAGTG	CACTATTCCATCCACAACG	NS4B	7342	7360	85.81
CATTGGAGTCAGCAATAGA	TCTATTGCTGACTCCAATG	Envelope protein E	985	1003	85.43
TGAACCAGCCAGAATTGCA	TGCAATTCTGGCTGGTTCA	NS4A	6751	6769	85.42
CAGCTGTGAGGGTAGTAGA	TCTACTACCCTCACAGCTG	NS2A	4143	4161	85.06
GCCCTGGCTTCGTGTCTTC	GAAGACACGAAGCCAGGGC	NS2A	3854	3872	85.02
GAGGCATTGGTAGAATTCA	TGAATTCTACCAATGCCTC	Envelope protein E	1682	1700	84.66
CATGGTGTGGAATAGAGTG	CACTCTATTCCACACCATG	NS5	10090	10108	84.61
GTGCATGGCTCCCAGCATA	TATGCTGGGAGCCATGCAC	Envelope protein E	1403	1421	84.52
CAAAGTGCAGGAGGTGAGA	TCTCACCTCCTGCACTTTG	NS5	7939	7957	84.28
GAAGATGGTCCACCCATGA	TCATGGGTGGACCATCTTC	NS2B	4475	4493	84.19
AGCTATGTGGTCTCGGGAA	TTCCCGAGACCACATAGCT	NS2B	4331	4349	84.12

Table S5. Predicted potential siRNA suppressors for Zika virus using DesiRm web server.

siRNA forward strand::reverse complementary	DesiRm predicted efficacy	imRNA SVMscore forward strand	imRNA SVMscore reverse strand
TTGATCTGGAGAATGAAGC::GCTTCATTCTCCAGATCAA	1.03	0.50	0.78
TCAATGGACATTGATCTGC::GCAGATCAATGTCCATTGA	0.99	0.72	0.87
TCTCAATGGACATTGATCT::AGATCAATGTCCATTGAGA	0.96	0.64	0.78
TAAAAAAGAGACTCCGGAC::GTCCGGAGTCTCTTTTTTA	0.96	1.09	1.12
TAAAAATGGACAAGCTTAG::CTAAGCTTGTCCATTTTTA	0.95	1.00	0.87
TTCTCAATGGACATTGATC::GATCAATGTCCATTGAGAA	0.94	0.68	0.77
TTTGATCTGGAGAATGAAG::CTTCATTCTCCAGATCAAA	0.88	0.55	0.81
TTGAACACAAAGAATGGAT::ATCCATTCTTTGTGTTCAA	0.86	0.83	0.95
TGAACACAAAGAATGGATC::GATCCATTCTTTGTGTTCA	0.86	0.67	0.92
ACAAAATGGACAGACATTC::GAATGTCTGTCCATTTTGT	0.86	0.50	0.66
TCATTTTCAGAGCCAATTG::CAATTGGCTCTGAAAATGA	0.85	0.56	0.57
TTGAAGAAGAGAATGACCA::TGGTCATTCTCTTCTTCAA	0.85	0.72	1.34
AAAAAGAGACTCCGGACAG::CTGTCCGGAGTCTCTTTTT	0.85	0.79	0.93
TTGAAGCAAGAATGCTTCT::AGAAGCATTCTTGCTTCAA	0.84	0.56	0.67
AACAAAAAATGACATAGCT::AGCTATGTCATTTTTTGTT	0.84	0.99	0.76
AAACAAAAAATCAAGAGTG::CACTCTTGATTTTTTGTTT	0.84	0.81	1.17
TTCATTTTCAGAGCCAATT::AATTGGCTCTGAAAATGAA	0.82	0.54	0.55
TGATCTGGAGAATGAAGCT::AGCTTCATTCTCCAGATCA	0.82	0.56	0.85
ATTGAAAGTGAAAAGAATG::CATTCTTTTCACTTTCAAT	0.81	0.55	0.75
AAGTTTGATCTGGAGAATG::CATTCTCCAGATCAAACTT	0.81	0.54	0.61
ATAAAAAAGAGACTCCGGA::TCCGGAGTCTCTTTTTTAT	0.81	0.96	1.18
CAAAGAATGGATCTATCTC::GAGATAGATCCATTCTTTG	0.81	0.53	0.83
TAGCTATCATTCTGCTTGT::ACAAGCAGAATGATAGCTA	0.81	0.75	0.55
CCAACTTCAAGGCTGACCG::CGGTCAGCCTTGAAGTTGG	0.80	0.64	0.63
